# Supplementary material for: Ectoparasite survey of schoolchildren in the Republic of Guinea
Source: PLOS Glob Public Health. 2026 Jan 13;6(1):e0005496. doi: 10.1371/journal.pgph.0005496 (PMC12799003; doi:10.1371/journal.pgph.0005496)
Supplement: S1 Table — (DOCX) [file pgph.0005496.s001.docx]

| Host  ID | Host Age | Nb of lice | ID of host | Sex of lice | Stage of lice | Tube ID | Commentaire  Haplotypes/ bacteria detection | | |
| --- | --- | --- | --- | --- | --- | --- | --- | --- | --- |
| SITE/KANKAN DIANKANA | | | | | | | **Haplogroupes** | ***Acinetobacter* spp.** | ***Acinetobacter baumanii*** |
| 1 | 11 | 1 | KD050 | F | L3 | 1 | E39 | 40.23 | NEG |
| 2 | 10 | 7 | KD075 | F  F  F  F  M  F  M | L3  L3  L3  L3  L3  L3  L3 | 2  3  4  5  6  7  8 | E77  E77  E77  E39  E39  E39  E39 | 37.97  38.78  37.69  NEG  39.78  37.44  38.20 | NEG  NEG  NEG  NEG  NEG  NEG  NEG |
| 3 | 11 | 1 | KD062 | F | L3 | 9 | E39 | NEG | NEG |
| 4 | 11 | 9 | KD048 | F  M  F  F  F  F  F  F  M | L2  L3  L3  L3  L3  L3  L3  L3  L3 | 10  11  12  13  14  15  16  17  18 | E39  E39  E39  E39  E39  E39  E39  E39  E39 | 37.48  36.87  NEG  NEG  NEG  37.41  36.82  37.26  NEG | NEG  27.41  NEG  NEG  NEG  NEG  NEG  NEG  NEG |
| 5 | 12 | 3 | KD053 | M  M  M | L3  L3  L3 | 19  20  21 | E39  E39  E39 | NEG  NEG  NEG | NEG  NEG  NEG |
| 6 | 11 | 9 | KD070 | F  F  M  F  M  F  M  M  M | L3  L3  L3  L3  L3  L3  L3  L3  L2 | 22  23  24  25  26  27  28  29  30 | E49  E49  E39  E49  E39  E39  E39  E39  E39 | 37.14  38.97  NEG  37.45  36.43  NEG  36.90  NEG  38.05 | NEG  NEG  NEG  NEG  38.34  NEG  NEG  NEG  36.04 |
| 7 | 11 | 3 | KD074 | F  M  M | L3  L3  L3 | 31  32  33 | E39  E39  E39 | NEG  38.46  38.05 | NEG  NEG  NEG |
| 8 | 10 | 4 | KD042 | F  F  M  M | L3  L3  L3  L2 | 34  35  36  37 | E39  E39  E39  E39 | NEG  NEG  40.90  38.43 | NEG  NEG  NEG  NEG |
| SITE/ BOKE GOREYE | | | | | | | | | |
| 9 | 5 | 5 | BG064 | F  M | L3  L3 | 38  39 | E39  E39 | NEG  NEG | NEG  NEG |
| 10 | 8 | 2 | BG060 | F | L3 | 40 | A17 | 32.11 | 35.22 |
| SITE /KANKAN BORDO 1 | | | | | | | | | |
| 11 | 11 | 11 | KB073 | M  M  M  M  M  M  F  F  M  M  F | L2  L2  L2  L3  L3  L3  L3  L3  L3  L3  L3 | 41  42  43  44  45  46  47  48  49  50  51 | E39  E39  E39  E39  E39  E39  E39  E39  E39  E39  E39 | NEG  NEG  NEG  NEG  NEG  NEG  NEG  NEG  37.42  38.01  36.84 | NEG  NEG  NEG  NEG  NEG  NEG  NEG  NEG  NEG  NEG  NEG |
| 12 | 11 | 3 | KB017 | M  F  M | L3  L3  L3 | 52  53  54 | E39  E39  E39 | NEG  NEG  37.62 | NEG  NEG  NEG |
| 13 | 10 | 1 | KB071 | M | L2 | 55 | E39 | 37.61 | NEG |
| 14 | 8 | 4 | KB067 | M  F  M  F | L3  L3  L3  L3 | 56  57  58  59 | E39  E39  E39  E39 | 37.94  37.24  NEG  NEG | NEG  NEG  NEG  NEG |
| 15 | 8 | 6 | KB059 | M  M  M  M  X  X | L3  L3  L1  L1  NA  NA | 60  61  62  63  64  65 | E39  E39  E39  A17  NA  NA | NEG  NEG  NEG  NEG  NEG  NEG | NEG  NEG  NEG  NEG  NEG  NEG |
| 16 | 7 | 1 | KB044 | M | L3 | 66 | E39 | NEG | NEG |
| 17 | 11 | 7 | KB026 | M  M  M  M  M  F  M | L3  L3  L3  L3  L3  L3  L3 | 67  68  69  70  71  72  73 | E39  E39  E39  E39  E39  E39  E39 | 37.16  37.21  NEG  37.47  NEG  29.93  NEG | NEG  NEG  NEG  NEG  NEG  NEG  NEG |
| 18 | 9 | 8 | KB074 | F  F  M  M  F  M  M  X | L3  L3  L3  L3  L3  L3  L2  L1 | 74  75  76  77  78  79  80  81 | E39  E39  E39  E39  E39  E39  E39  E39 | NEG  36.35  NEG  NEG  NEG  38.01  NEG  NEG | NEG  NEG  NEG  NEG  NEG  NEG  NEG  NEG |


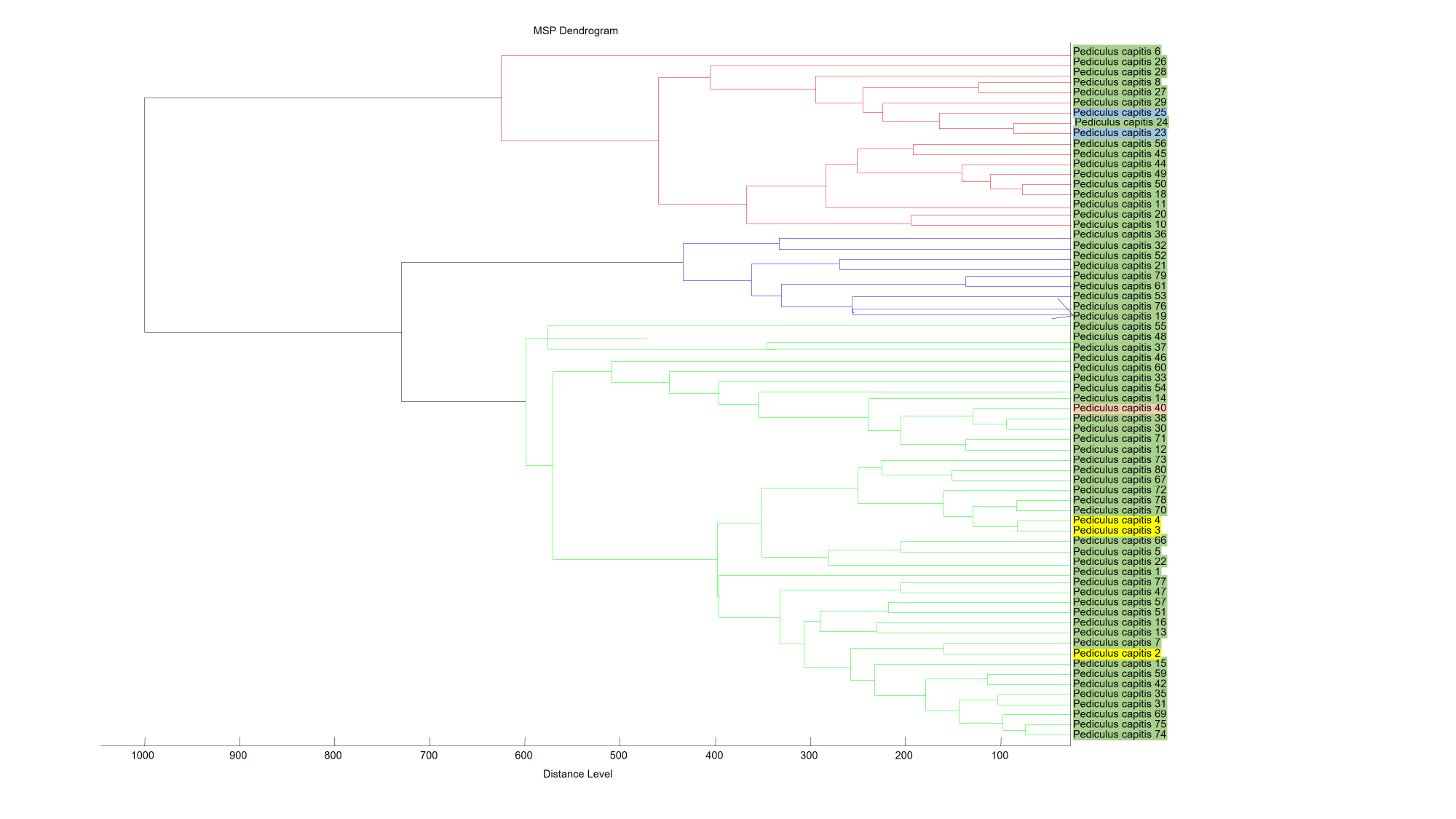


**S1 Figure**. MSP dendrogram based on MALDI-TOF MS spectra of cephalothoraxes from haplotypes E39 (green), E49 (blue), E77 (yellow), and A17 (pink). Distance units represent the relative similarity between MS spectra. The dendrogram was generated using Biotyper v3.0 software. Abbreviation: MSP, main spectrum profile.
