# Supplementary material for: Ectoparasite survey of schoolchildren in the Republic of Guinea
Source: PLOS Glob Public Health. 2026 Jan 13;6(1):e0005496. doi: 10.1371/journal.pgph.0005496 (PMC12799003; doi:10.1371/journal.pgph.0005496)
Supplement: S1 File — (DOCX) [file pgph.0005496.s002.docx]

Inclusivity in global research

PLOS’ policy on inclusivity in global research aims to improve transparency in the reporting of research performed outside of researchers’ own country or community and ensures that PLOS publications reporting global research adhere to high standards for research ethics and authorship. Authors of relevant research articles may be asked to complete the questionnaire below, which outlines ethical, cultural, and scientific considerations specific to inclusivity in global research. This questionnaire may be requested when researchers have travelled to a different country to conduct research, if research uses samples collected in another country, research with Indigenous populations or their lands, or if research is on cultural artefacts. Researchers travelling to another country solely to use laboratory equipment will not normally be required to complete the questionnaire. However, the questionnaire can be requested at the journal’s discretion for any submission – if you have been requested to complete this questionnaire by the PLOS journal you submitted to, please do so.

Please complete the questionnaire below and include this as a Supporting Information file with your manuscript. Note that if your paper is accepted for publication, this checklist will be published with your article in the supporting information files. Please ensure that you reference the checklist in the main body of your manuscript. We suggest adding a subsection ‘Inclusivity in global research’ to your Methods section and adding the following sentence: “Additional information regarding the ethical, cultural, and scientific considerations specific to inclusivity in global research is included in the Supporting Information (SX Checklist)”

The questions have been designed to be applicable to a wide range of study types, and there are subsections for both human subjects research and non-human subjects research. If any of the questions are not relevant to your research please mark them as “N/A” as appropriate.

**Ethical considerations, permits and authorship**

*This section is applicable to all research types.*

Provide details as to who granted permissions and/or consent for the study to take place in the Methods section of your manuscript. This should include the names of **all** ethics boards, governmental organizations, community leaders or other bodies that provided approval for the study. If individuals provided approval refer to these people by their role or title but do not list their name(s).

Only randomized primary school children whose parents or guardians provided informed consent were included in the study. Participating children also gave written informed consent. Only those exhibiting symptoms of head lice and/or scabies were sampled. Children who declined to participate, along with their parents or legal representatives, were non-included in the study.

The study protocol was approved by the National Health Research Ethics Committee of the Republic of Guinea (No. 041/CNERS/23).

Education managers, school directors, and village elders accompanied the researchers throughout the study.

If there were any deviations from the study protocol after approval was obtained please provide details of these changes in the Methods section of your manuscript.
Did this study involve local collaborators that are residents of the country where the research was conducted or members of the community studied? If you do not have any authors from said communities, please provide an explanation for this below.

Yes, this study involved local collaborators. The second author is a native of the community studied and serves as the co-supervisor of this doctoral thesis. His deep understanding of the local context and direct involvement were essential throughout the research process, from study design to fieldwork.

Reported on page number: N/A

Everyone listed as an author should meet PLOS’ criteria for authorship and all individuals who meet these criteria should be included in the author byline, rather than the acknowledgements. For further information please see the journal’s Authorship Policy.

**Human subjects research (e.g. health research, medical research, cross-cultural psychology)**

Did you obtain written informed consent from a representative of the local community or region before the research took place? How did you establish who speaks for the community? Details of written informed consent obtained from study participants should be reported separately in the Methods section of your manuscript.

This study was conducted in accordance with national and institutional ethical guidelines. The research protocol was approved by the relevant public health and education authorities in Guinea, including the national office for preschool health and the Ministry of Pre-University Education.

Before any fieldwork was conducted, oral consent was obtained from local authorities and community representatives, including prefectural directors of education, school principals, presidents of the parents’ associations (APAEs), and local community leaders.

At the local level, we sought and obtained **oral consent** from key community representatives prior to any data collection at each study site. These included the **prefectural directors of education, the heads of each school, the presidents of the students’ parents associations (APAEs), and local community leaders**. These individuals were identified as legitimate representatives of the communities based on their official roles and social responsibilities within the school and local governance structures.

We acknowledge that no written consent was obtained at the community level; however, oral consent was deemed appropriate and culturally acceptable in this context, and was granted following clear explanations of the study’s objectives and procedures. Written informed consent from individual participants is addressed separately in the Methods section of the manuscript.

How did members of the local community provide input on the aims of the research investigation, its methodology, and its anticipated outcome(s)?

The community leaders and organizations gave us thier feedback on the feasibility and appropriateness within the local context of our styudy and facilitated trust and ensured that the research aligned with community needs and values.

When engaging with the local community, how did you ensure that the informed consent documents and other materials could be understood by local stakeholders?

We used clear and straightforward language, avoiding technical jargon that may not be familiar to community members. Participants’ information was translated into local languages to ensure proper informed consent.

Will the findings of the research be made available in an understandable format to stakeholders in the community where the study was conducted (e.g. via a presentation, summary report, copies of publications, etc.)? Please provide details of how this will be achieved.

Yes, the findings of this research will be shared in an accessible and understandable format with relevant stakeholders in the community where the study was conducted. The results will first be formally presented to the **School Health Division of the Ministry of Pre-University Education**, which is the national body responsible for school-based health initiatives, delegated by the Ministry of Health.

In collaboration with this division, and depending on available resources, we also plan to organize **awareness campaigns** in the local communities to educate parents, teachers, and schoolchildren about the public health importance of the diseases studied.

The dissemination will include:

- A summary report in French tailored for non-scientific audiences;
- Community feedback sessions or meetings in selected schools;
- Copies of any resulting publications or policy briefs shared with local education and health authorities.

These steps aim to ensure that the research findings contribute not only to academic knowledge but also to **local health awareness and decision-making**.

**Non-human subjects research using specimens/ animals collected as part of the study, or those housed in archival collections. Examples include archaeology, paleontology, botany and zoology.**

Did the permission you obtained from a local authority to perform the study include an agreement on access to outputs and benefit sharing? This may include procedures to enable fair distribution of the benefits and resources arising from the research performed. Please include any details of Prior Informed Consent and Benefit Sharing Agreements obtained. These may be required by field-specific regulations, for example the Convention on Biological Diversity (CBD) and the associated Nagoya Protocol.

N/A

If the material used in your study was imported, please A) provide the year it was imported and B) indicate whether permits were obtained to import/export the materials used, C) provide details of any permits obtained. If this information is not available, please indicate this.

**A)** The biological samples used in this study were imported in **2023**.

**B)** Yes, appropriate authorizations were obtained for the transfer of the samples. A **Material Transfer Agreement (MTA)** was signed to govern the export and use of the materials collected during the study.

**C)** The MTA was established between the **National Institute of Public Health of Guinea (INSP)**—the institution of origin—and the **Fondation Méditerranée Infection** in Marseille, France, where the laboratory analyses were conducted (Mycology and Parasitology Laboratory).

In addition, a **specific export authorization** was issued by the Fondation Méditerranée Infection for the **transport and analysis of lice specimens**, which were part of a complementary investigation related to the study. All transfers were carried out in compliance with biosafety and ethical standards, and the materials were used strictly for non-commercial research purposes.

Copies of the MTA and export documentation are available upon request.

If you used archival specimens, please state how the material used in your study was acquired by the institute it is held in and provide details of any permits obtained for the original excavations/ sample collection. If this information is not available, please indicate this.

N/A

How was the potential cultural significance of the materials collected in your study to local communities considered in your research design? Were Indigenous peoples and/or local researchers and institutions involved with archaeological excavations / collection of specimens? If so, please provide a description of their involvement.

We collaborated with local institutions to achieve a clear understanding of the cultural significance. Local researchers advised the research team on using respectful practices and appropriate methodologies.

If your manuscript includes photographs of human remains please indicate whether authors obtained permission from descendants or affiliated cultural communities to do so.

N/A
