# Supplementary material for: Ectoparasite survey of schoolchildren in the Republic of Guinea
Source: PLOS Glob Public Health. 2026 Jan 13;6(1):e0005496. doi: 10.1371/journal.pgph.0005496 (PMC12799003; doi:10.1371/journal.pgph.0005496)
Supplement: Attachment — Submitted filename: Authors Response 03.06.2025.docx [file pgph.0005496.s003.docx]

**Journal Requirements:**

1. Please include a complete copy of PLOS’ questionnaire on inclusivity in global research in your revised manuscript. Our policy for research in this area aims to improve transparency in the reporting of research performed outside of researchers’ own country or community. The policy applies to researchers who have travelled to a different country to conduct research, research with Indigenous populations or their lands, and research on cultural artefacts. The questionnaire can also be requested at the journal’s discretion for any other submissions, even if these conditions are not met.  Please find more information on the policy and a link to download a blank copy of the questionnaire here: [https://journals.plos.org/globalpublichealth/s/best-practices-in-research-reporting](https://urldefense.com/v3/__https:/journals.plos.org/globalpublichealth/s/best-practices-in-research-reporting__;!!JQ5agg!dEEeMqxrToRjy85TyvUa2VpAUs-lYGh2xrXTyuJ4aiOIhVBRrRqvj0biE9LRh4GoEayRKxJ7IODDkWFGS4jYhEM$). Please upload a completed version of your questionnaire as Supporting Information when you resubmit your manuscript.

**Authors’ Response:** Done

2. We have amended your Competing Interest statement to comply with journal style. We kindly ask that you double check the statement and let us know if anything is incorrect.

**Authors’ Response:** OK.

3. We do not publish any copyright or trademark symbols that usually accompany proprietary names, eg (R), (C), or TM  (e.g. next to drug or reagent names). Please remove all instances of trademark/copyright symbols throughout the text, including ®, ™ on page 8, 9, 13.

**Authors’ Response:** We thank the editor for its comment. The remarks have been duly taken into account, and the names in question have been removed from the final document on pages 8, 9, and 14, line: 214-234-286-287-289-290.

4. Some material included in your submission may be copyrighted. According to PLOS’s copyright policy, authors who use figures or other material (e.g., graphics, clipart, maps) from another author or copyright holder must demonstrate or obtain permission to publish this material under the Creative Commons Attribution 4.0 International (CC BY 4.0) License used by PLOS journals. Please closely review the details of PLOS’s copyright requirements here: PLOS Licenses and Copyright. If you need to request permissions from a copyright holder, you may use PLOS's Copyright Content Permission form.

Please respond directly to this email or email the journal office and provide any known details concerning your material's license terms and permissions required for reuse, even if you have not yet obtained copyright permissions or are unsure of your material's copyright compatibility.

Potential Copyright Issues:

a. Figure 1: please (a) provide a direct link to the base layer of the map (i.e., the country or region border shape) and ensure this is also included in the figure legend; and (b) provide a link to the terms of use / license information for the base layer image or shapefile. We cannot publish proprietary or copyrighted maps (e.g. Google Maps, Mapquest) and the terms of use for your map base layer must be compatible with our CC-BY 4.0 license.

Note: if you created the map in a software program like R or ArcGIS, please locate and indicate the source of the basemap shapefile onto which data has been plotted.

If your map was obtained from a copyrighted source please amend the figure so that the base map used is from an openly available source. Alternatively, please provide explicit written permission from the copyright holder granting you the right to publish the material under our CC-BY 4.0 license.

Please note that the following CC BY licenses are compatible with PLOS license: CC BY 4.0, CC BY 2.0 and CC BY 3.0, meanwhile such licenses as CC BY-ND 3.0 and others are not compatible due to additional restrictions.

If you are unsure whether you can use a map or not, please do reach out and we will be able to help you. The following websites are good examples of where you can source open access or public domain maps:

* U.S. Geological Survey (USGS) - All maps are in the public domain. ([http://www.usgs.gov](https://urldefense.com/v3/__http:/www.usgs.gov__;!!JQ5agg!dEEeMqxrToRjy85TyvUa2VpAUs-lYGh2xrXTyuJ4aiOIhVBRrRqvj0biE9LRh4GoEayRKxJ7IODDkWFGzssHtFc$))

* PlaniGlobe - All maps are published under a Creative Commons license so please cite “PlaniGlobe, [http://www.planiglobe.com](https://urldefense.com/v3/__http:/www.planiglobe.com__;!!JQ5agg!dEEeMqxrToRjy85TyvUa2VpAUs-lYGh2xrXTyuJ4aiOIhVBRrRqvj0biE9LRh4GoEayRKxJ7IODDkWFGCrDg11E$), CC BY 2.0” in the image credit after the caption. ([http://www.planiglobe.com/?lang=enl](https://urldefense.com/v3/__http:/www.planiglobe.com/?lang=enl__;!!JQ5agg!dEEeMqxrToRjy85TyvUa2VpAUs-lYGh2xrXTyuJ4aiOIhVBRrRqvj0biE9LRh4GoEayRKxJ7IODDkWFGR-NRdHA$))

* Natural Earth - All maps are public domain. ([http://www.naturalearthdata.com/about/terms-of-use/](https://urldefense.com/v3/__http:/www.naturalearthdata.com/about/terms-of-use/__;!!JQ5agg!dEEeMqxrToRjy85TyvUa2VpAUs-lYGh2xrXTyuJ4aiOIhVBRrRqvj0biE9LRh4GoEayRKxJ7IODDkWFGwgfIyfw$))

Additional Editor Comments (if provided):

Based on the reviews received, I have decided for the manuscript to undergo a 'Major Revision'.


[Note: HTML markup is below. Please do not edit.]

**Figure 1#:**

**Authors’ Response:**

We would like to clarify that the map presented in Figure 1 was created entirely from primary data collected in the field using the OSMTracker application. During our visits to the study sites, GPS coordinates were recorded, then exported to Excel, and subsequently imported into QGIS for map design and visualization (as stated line:110). No external basemap or shapefile (such as Google Maps, MapQuest, or similar sources) was used in the creation of this figure. The map is based solely on our own field-collected data and does not include any copyrighted or proprietary mapping layers. Accordingly, the figure fully complies with the journal’s CC-BY 4.0 licensing requirements.

**5. Review Comments to the Author**

Reviewer #1: I appreciate the efforts undertaken in this ectoparasite survey. I have a few comments and suggestions that could help further strengthen the manuscript:
1. Abstract: Please mention the creation/expansion of the MALDI-TOF MS database for lice

**Authors' Response:** We thank the reviewer for this insightful comment. In response, we have updated the abstract to include the creation of the MALDI-TOF MS reference database for lice, as well as the fact that PCR testing for scabies was negative in all samples. The revised section now reads as follows: “*Analysis of the MS spectra revealed that 70 (87%) were of acceptable quality. Five of these spectra were used to establish the MALDI-TOF MS reference database. Blind testing of the remaining 65 spectra successfully identified them as* Pediculus humanus capitis*, with log score values (LSVs) ranging from 1.708 to 2.339.*” Line 26-29.

and the fact that scabies PCR was negative in all samples should be mentioned in the abstract, as these are key findings.

**Authors' Response:** we added the following sentence: “PCR testing for Sarcoptes scabiei was negative in all scabies-like lesion samples”.

2. Methods

i. Please specify how many spectra were collected per specimen for library preparation, as reproducibility of MS spectra is critical.

**Authors' Response:** As stated in section 6.2 “*Sample Loading on MALDI-TOF Target Plates”*, one microliter (1 µL) of supernatant from each sample was spotted in quadruplicate onto the MALDI-TOF MS target plate. Accordingly, for the construction of the database, the four spectra obtained from each of the five selected specimens were used. We have clarified this in the manuscript by adding the following sentence (Lines 162-169): “*The MALDI-TOF MS reference database was constructed using the four MS spectra acquired from the cephalothorax of five lice specimens with the MALDI-Biotyper software (Bruker Daltonics).*”

ii. Clarify what gold standard was used for lice identification for MALDI-TOF library creation. It appears PCR confirmation was used — please explicitly state this in the methods section.

**Authors' Response:** The identification of the lice species used to generate the spectra for the MALDI-TOF MS reference database was confirmed by standard PCR followed by sequencing, targeting the mitochondrial *cytb* gene. We have clarified this point in the manuscript by adding the following sentence (Lines 196-199): “*The five lice specimens whose MS spectra were selected for the construction of the MALDI-TOF MS reference database had been identified by analysing the nucleotide sequence of a 347-bp fragment of the mitochondrial cytb gene.*”

iii. Cluster analysis (MSP dendrogram) could be performed to illustrate how the lice specimens relate to each other, are the halplotypes differentiated on MALDI-TOF?

**Authors' Response:** We thank the reviewer, this analysis was conducted; however, since it did not produce a clear separation of the louse specimens according to haplotypes, the results were initially not included in the manuscript. Nevertheless, as you rightly pointed out, we have now decided to include this analysis in the manuscript as a follow-up :

- In Materials and methods section 6.4. MALDI-TOF MS spectra analysis “MALDI-TOF MS spectra were visually inspected using FlexAnalysis v3.3 software (Bruker Daltonics, Bremen, Germany). Subsequently, the spectra were processed using ClinProTools v2.2 and MALDI Biotyper v3.0 (Bruker Daltonics, Bremen, Germany) for data analysis, including spectral smoothing, baseline subtraction, and peak detection. The reproducibility of the MS spectra was evaluated by comparing the main spectral profiles (MSPs) obtained from four technical replicates (spots) of each sample using MALDI Biotyper v3.0. Furthermore, cluster analysis (dendrogram) was performed to evaluate the ability of MALDI-TOF MS to discriminate between spectra corresponding to different louse haplotypes”. Line 186-194
- In the results section 3- MALDI-TOF MS lice identification “To investigate the potential of MALDI-TOF MS for discriminating between different louse haplotypes, a cluster analysis (dendrogram) was performed using the main spectral profiles (MSPs). However, the resulting dendrogram did not clearly segregate spectra according to haplotype. This suggests that the protein profiles generated under the current experimental conditions are not sufficiently discriminatory at the haplotype level (Supplementary Figure 1). ” Line 329-334
- In discussion section “These results are consistent with previous reports indicating that, while MALDI-TOF mass spectrometry is highly effective for species-level identification of arthropods, its resolution for detecting intraspecific genetic variation such as mitochondrial haplotypes remains limited (ref 42,43,44) (PMID: 24423215, PMID: 25957576, PMID: 30390691). Further optimization strategies, including the targeting of specific protein fractions, the integration of MALDI-TOF MS with molecular approaches, or the development of more advanced analytical software, may enhance the discriminatory power of this technique at the subspecies or haplotype level in future studies.” Line 390-397

iv. In the discussion, kindly mention previous studies where MALDI-TOF MS has been applied for arthropod (especially lice) identification and compare findings where relevant.

**Authors’ response:** We thank the reviewer for their valuable suggestion. As requested, we have included a paragraph discussing previous studies in which MALDI-TOF MS has been employed for the identification of arthropods, with a particular focus on lice. We also provide a comparative analysis of our findings in relation to these earlier works as follows.

Numerous investigations have substantiated the efficacy, rapidity, precision, and cost-efficiency of matrix-assisted laser desorption/ionization time-of-flight mass spectrometry (MALDI-TOF MS) in the fields of medical and veterinary entomology (ref 45,46,47)(PMID: 27070074, PMID: 33733821, PMID: 36422371). Over the past decade, MALDI-TOF MS has been increasingly proposed as a reliable alternative for the taxonomic identification of various arthropod families, including lice (ref 48,20,7) (PMID: 37887837, PMID: 33346344, PMID: 32351208). The findings of our study corroborate the effectiveness of MALDI-TOF MS for the identification of lice, including specimens preserved in alcohol, as previously reported (ref 20,7) (PMID: 33346344, PMID: 32351208). Nevertheless, a limitation of our work lies in its focus on a single louse species, whereas earlier studies have encompassed multiple louse species, allowing for broader comparative analyses (ref 20,7) (PMID: 33346344, PMID: 32351208). Line 398-408

v. The stability of cephalothorax MS profiles can be affected by alcohol storage. Ideally, lice should be dried at room temperature for at least 12 hours to avoid alcohol residue affecting MS spectra. You have mentioned drying at room temperature for only 5 minutes, which could be insufficient. Please discuss this as a potential limitation. Suggested reference: Benyahia, H.; Parola, P.; Almeras, L. Evolution of MALDI-TOF MS Profiles from Lice and Fleas Preserved in Alcohol over Time. Insects 2023, 14, 825. [https://doi.org/10.3390/insects14100825](https://urldefense.com/v3/__https:/doi.org/10.3390/insects14100825__;!!JQ5agg!dEEeMqxrToRjy85TyvUa2VpAUs-lYGh2xrXTyuJ4aiOIhVBRrRqvj0biE9LRh4GoEayRKxJ7IODDkWFG-N90uvg$)

**Authors’ response:** We thank the reviewer, However, the phrase “dry at room temperature for 5 minutes” refers to the preparation of the lice prior to dissection. To ensure the removal of alcohol residues from the cephalothorax, it is clearly stated in section 6, *MALDI-TOF MS analysis of lice* (6.1. Pretreatment), that: “Cephalothoraxes were then incubated in a dry heat bath at 37 °C overnight to remove residual organic solvents.” Line 159-160

3.Results
i. You mention that five lice were identified after library preparation in a blind test. Please include the results of this blind testing

**Authors’ response:** We thank the reviewer, However, unless we are mistaken, the results of the blind test are indeed reported in the manuscript as follows: “*Blind testing of the remaining 65 spectra (not included in the MALDI-TOF MS database) against our database identified them all as Pediculus humanus capitis, with MALDI Biotyper log scores values ranging from 1.708 to 2.339 with a mean of 2.05.*” Line 325-326.

However, if this wording may lead to confusion, we have revised the sentence as follows: “*The remaining 65 spectra (i.e., those not included in the MALDI-TOF MS database) were blindly tested against the MALDI-TOF MS database. All were identified as* *Pediculus humanus capitis*, *with MALDI Biotyper log score values ranging from 1.708 to 2.339 (mean: 2.05).*”Line 326-329.

Reviewer #2: 1. Could you explain why block randomisation was used in an observational study or stratified randomisation was used, and it is misrepresented as block randomisation?

**Authors’ response:** We agree with the reviewer’s remark. What we have done is rather “stratified” randomisation. We have corrected this in the manuscript (line 134).

2. Line 293: Any hypothesis why males were not infested with Lice?

**Authors’ response:** We thank the Reviewer for this pertinent remark. As mentioned in the manuscript, no lice infestation was recorded among boys in this study. A likely explanation is that prepubescent boys in the studied population typically have their heads shaved, which likely reduces or prevents lice infestation.

3. Line 259: Kindly introduce Bartonelle quintana as B. quintana before using the short form.

**Authors’ response:** We thank the Reviewer for this relevant remark. We have taken it into account and have now introduced *Bartonella quintana* in full before using the abbreviated form *B. quintana* in the revised manuscript (line 71-72).

4. Line 346: Before using the short form, kindly introduce Yersinia pestis as Y. pestis.

**Authors’ response:** We thank the Reviewer for this observation. We have addressed this point by introducing *Yersinia pestis* in full before using the abbreviated form *Y. pestis* in the revised manuscript (line 362).

4. Line 352: Kindly introduce Acinetobacter baumannii as B. quintana A. baumannii before using the short form.

**Authors’ response:** We thank the Reviewer for this helpful suggestion. We have corrected the text by introducing *Acinetobacter baumannii* in full before using the abbreviated form *A. baumannii* in the revised manuscript (line 368).

5. After introducing the short form, please italicise the short form. Do not randomly use the complete, abbreviated, and short forms for organisms.

**Authors’ response:** We thank the Reviewer for this valuable observation. We have carefully revised the manuscript to ensure that, after introducing the full name of each organism, the abbreviated forms are used consistently and italicised as required. We have also avoided alternating arbitrarily between full and abbreviated forms throughout the text

6. Kindly mention and elaborate which clades of Pediculus humanus capitis are having *Acinetobacter baumannii*.

**Authors’ response:** We thank the Reviewer for this relevant question. Among the samples tested, only three were positive for *Acinetobacter baumannii*, and all belonged to clade E of *Pediculus humanus capitis*. However, further research is needed to assess whether this association is clade-specific or is due to the limited sample size.
