# Supplementary material for: Ectoparasite survey of schoolchildren in the Republic of Guinea
Source: PLOS Glob Public Health. 2026 Jan 13;6(1):e0005496. doi: 10.1371/journal.pgph.0005496 (PMC12799003; doi:10.1371/journal.pgph.0005496)
Supplement: Attachment — Submitted filename: Response to Reviewers _ectoparasites_2025.09.23.docx [file pgph.0005496.s004.docx]

Reviewer #3: The use of MALDI for the identification of ticks, mosquito and lice are well standardized. The study is well-designed. I have a couple of queries as mentioned below:

1. Whether there was any database available for the lice identification?
   **Auteurs response:** We thank the reviewer for their comment. Yes, a database for lice identification was available in our laboratory. However, it only includes body lice and animal lice, the species of which are reported in articles indexed in PubMed (PMID: 33346344; PMID: 32351208). For clarity, the sentence has been added in Section **6.5**, Reference Spectra Database Construction ‘This database already contains reference spectra for multiple arthropod species, including human body lice and domestic animal lice (*1- Benyahia H, Ouarti B, Diarra AZ, Boucheikhchoukh M, Meguini MN, Behidji M, Benakhla A, Parola P, Almeras L. Identification of Lice Stored in Alcohol Using MALDI-TOF MS. J Med Entomol. 2021 May 15;58(3):1126-1133. doi:10.1093/jme/tjaa266 ; 2- Ouarti B, Laroche M, Righi S, Meguini MN, Benakhla A, Raoult D, Parola P. Development of MALDI-TOF mass spectrometry for the identification of lice isolated from farm animals. Parasite. 2020; 27:28. doi: 10.1051/parasite/2020026*).
2. If not then, why the used 5 spectra for database creation,on what basis ?, possibly DNA sequence based analysis, need to get clear clarifications .

**Auteurs response:** We thank the reviewer for their comment. The spectra of the five specimens were not selected based on DNA sequences but rather following the construction of a dendrogram. Representative specimens were chosen according to their clustering patterns to ensure optimal representativeness. Subsequently, their identities were confirmed by sequencing. This procedure was incorporated into Section **6.5**, Reference Spectra Database Construction, as follows: ‘Based on both molecular data and the appearance of the MS spectra, five representative head lice specimens were selected for inclusion in the MALDI-TOF MS reference database. For each specimen, four high-quality spectra were acquired and used to generate main spectrum profiles (MSPs), following confirmation of their morphological identification by molecular analysis. The reference database was constructed using MALDI-Biotyper v3.0 software (Bruker Daltonics, Bremen, Germany). MSPs were created using an unbiased algorithm that integrates peak position, intensity, and frequency information. This database already contains reference spectra for multiple arthropod species, including human body lice and domestic animal lice (*1-Benyahia H, Ouarti B, Diarra AZ, Boucheikhchoukh M, Meguini MN, Behidji M, Benakhla A, Parola P, Almeras L. Identification of Lice Stored in Alcohol Using MALDI-TOF MS. J Med Entomol. 2021 May 15;58(3):1126-1133. doi:10.1093/jme/tjaa266; 2-Ouarti B, Laroche M, Righi S, Meguini MN, Benakhla A, Raoult D, Parola P. Development of MALDI-TOF mass spectrometry for the identification of lice isolated from farm animals. Parasite. 2020;27:28. doi: 10.1051/parasite/2020026*).

3. When they created database,how many spectra of the same sample was captured ? Usualy 12-14 spots spectra captured and best 10 out of 14 spectra is selected for creating database, whether they follow the same.

**Auteurs response:** We thank the reviewer for their comment. In our laboratory, four spectra from each specimen were combined to generate a main spectrum profile (MSP) using MALDI-Biotyper v3.0 software (Bruker Daltonics, Bremen, Germany). This has been clarified as follows see below

4. It is not clear, after the database creation,what is the 70 spectra identification results.

**Auteurs response**: We thank the reviewer for their comment. We have modified this paragraph as follows: A total of 79 lice specimens were subjected to MALDI-TOF MS analysis. High-quality MS spectra defined as spectra with an intensity greater than 3000 arbitrary units and minimal background noise, were obtained for 70 of the 79 specimens (87%). For database construction, four MS spectra from five specimens, whose morphological identifications were confirmed by molecular analysis, were used to generate main spectrum profiles (MSPs) and update our MALDI-TOF MS reference database. A representative MS spectrum from each of these five specimens is shown in **Figure 3**. Blind testing of the MS spectra from the remaining 65 specimens, which were not included in the updated database, correctly identified all as *Pediculus humanus capitis*, with log score values ranging from 1.708 to 2.339 (mean: 2.05).’
